# The Discrimination between Syphilis and Scrofula

**Published:** 1871-03

**Authors:** 


### THE DISCRIMINATION BETWEEN SYPHILIS AND SCROFULA.

F. F. Maury, M. D., Surgeon to the Philadelphia Hospital (*Med. and Surg. Reporter*), in a lecture on "Infantile Syphilis," states that the parallelism between congenital syphilis and scrofula, when closely traced, so approximates that no human being can discriminate between them. He also believes that the unfolding of the next twenty-five years will establish the identity between so-called scrofula and inherited or quarternary syphilis, and this pathway leads him to the broad field of legalized prostitution, of which he is an advocate.

